# Muscle activity variability patterns and stride to stride fluctuations of older adults are positively correlated during walking

**DOI:** 10.1038/s41598-023-47828-9

**Published:** 2023-11-25

**Authors:** Sofia Jordão, Nick Stergiou, Rita Brandão, Pedro Pezarat-Correia, Raúl Oliveira, Nelson Cortes, João R. Vaz

**Affiliations:** 1 CIPER, Neuromuscular Research Lab, Faculty of Human Kinetics, University of Lisbon, Lisbon, Portugal; 2 Egas Moniz Center for Interdisciplinary Research (CiiEM), Egas Moniz School of Health & Science, Campus Universitário, Quinta da Granja, Monte da Caparica, 2829 – 511 Caparica, Portugal; 3 Hospital da Ordem Terceira Chiado, Lisbon, Portugal; 4 Department of Biomechanics, University of Nebraska at Omaha, Omaha, NE USA; 5 Department of Physical Education and Sport Science, Aristotle University, Thessaloniki, Greece; 6 School of Sport, Rehabilitation and Exercise Sciences, University of Essex, Colchester, UK; 7 Department of Bioengineering, George Mason University, Fairfax, VA USA

**Keywords:** Ageing, Neurophysiology, Complexity, Dynamical systems, Nonlinear dynamics

## Abstract

It has been found that fractal-like patterns are present in the temporal structure of the variability of healthy biological rhythms, while pathology and disease lead to their deterioration. Interestingly, it has recently been suggested that these patterns in biological rhythms are related with each other, reflecting overall health or lack of it, due to their interaction. However, the underlying neurophysiological mechanisms responsible for such dependency remain unknown. In addition, this relationship between different elements needs to be first verified before we even pursue understanding their interaction. This study aimed to investigate the relationship between two elements of the neuromuscular system, gait and muscle activity variability patterns in older adults. Twenty-one older adults walked at their preferred walking speed on a treadmill. Inter-stride intervals were obtained through an accelerometer placed on the lateral malleoli to assess the temporal structure of variability of stride-to-stride fluctuations. Inter muscle peak intervals were obtained through the electromyographic signal of the gastrocnemius to assess the temporal structure of the variability of the simultaneous muscle activity. The temporal structure of variability from both signals was evaluated through the detrended fluctuation analysis, while their magnitude of variability was evaluated using the coefficient of variation. The Pearson’s Correlation coefficient was used to identify the relationship between the two dependent variables. A significant strong positive correlation was found between the temporal structure of gait and muscle activity patterns. This result suggests that there is an interdependency between biological rhythms that compose the human neuromuscular system.

## Introduction

Gait variability is crucial for safe and adaptive human gait, and adequately interacting with a dynamic environment. Furthermore, a feature of gait variability, the temporal structure of stride-to-stride fluctuations could distinguish functional from dysfunctional locomotor systems^1^. This is because these fluctuations in healthy and functional gait exhibit a fractal-like pattern. Namely, they appear to be self-similar over multiple measurement scales. However, previous research has demonstrated that these patterns deteriorate due to ageing and pathology^2^. Specifically, older adults and neurological patients present alterations where there is increased randomness in the temporal structure of the stride-to-stride fluctuations^2–4^, suggesting decreased adaptability during walking. Physiologically, the fractal-like patterns that are present in health gait variability are considered an indicator of optimal connectivity between biological processes^5,6^. A breakdown in these patterns, as in the case of ageing, could also indicate a gradual decline or a decrease in the interactions between the elements that compose a given system^5,6^.

Recently, advances in the gait variability research have showed that healthy patterns can be restored in older adults^7,8^ and Parkinson’s Disease patients^9^. However, the current research lacks an understanding of the neurophysiological mechanisms that could be responsible for these changes in gait variability limiting translation to clinical practice. Considering that these fractal-like patterns emerge from an adequate connectivity and interaction within the system’s components, it is reasonable to question if the individual elements (e.g., muscles, motor unit recruitment, stretch reflexes, etc.) demonstrate similar patterns in their temporal structures. Recent preliminary research has showed such patterns to be present in the brain and heart when the individuals walked on a fractal-like path^10,11^ and the brain when they finger-thumb tapped coordinating with an auditory fractal-like stimulus^12^. These results suggest an interaction between elements and systems at the temporal structure domain. They also strengthen the importance of investigating if similar patterns, and changes due to pathology and aging, such as those present in the stride-to-stride fluctuations of gait variability patterns, are reflected in other elements of the neuromuscular system (e.g., muscle activity). This hypothesis is also supported by preliminary evidence from our laboratory, using electromyography, where we recently demonstrated that fluctuations in the temporal structure of the gastrocnemius muscle activity variability patterns, present similar properties to those found in gait (Vaz et al., *under review*). Specifically, we experimentally manipulated the gait patterns of healthy young individuals by asking them to synchronize their gait to different temporally structured metronomes. We have shown a strong positive correlation indicating that when gait variability patterns become more random, muscle activity variability patterns also present increased randomness; and when gait variability patterns become more organized and fractal-like, muscle activity variability patterns demonstrate similar effects. These findings indicate that the temporal structure of gait and muscle activity variability patterns are likely to be interconnected. The next logical step is to investigate if aging or pathology affects muscle activity variability patterns in a similar fashion as gait variability patterns.

Therefore, the present study investigated if the temporal structure of gait variability patterns of older adults correlates with the temporal structure of the *gastrocnemius medialis* (GM) muscle activity variability patterns. We anticipated a positive significant correlation, supporting the rationale that there is a close interaction between the patterns of these elements that compose the human neuromuscular system. In addition, this study also investigated if the temporal structure of gait and muscle variability patterns differ. We hypothesized that no differences will be observed, since we anticipated that muscle activity variability patterns will reflect the patterns of the mechanical output.

## Methods

### Participants

Twenty-one participants (8 females and 13 males; 70.3 ± 4.5 years, 1.67 ± 0.07 m, 75.16 ± 11.7 kg) were included in this study. Twenty-one participants were determined, a priori*,* as the number needed to provide 85% power at an α = 0.05, considering a moderate correlation of 0.6. The participants were recruited by word of mouth from local health-related facilities (e.g., hospitals, clinics, senior residencies). Participants signed an informed consent previously approved by the Ethics Committee from the Faculty of Human Kinetics, University of Lisbon. All experimental protocols were approved by this Committee. For demographic purposes and inclusion criteria, they took on a general health assessment before they underwent the walking tests. Participants were excluded if they were younger than 65 years, suffered from a musculoskeletal or neurological condition that could affect gait and balance, or reported any cardiovascular clinical impairment. They had to be able to walk on a treadmill for 12 consecutive minutes and had to score > 24 on the Mini Mental State Examination (MMSE) for cognitive function to be eligible. All participants took on self-filling questionnaires related to quality of life and health status (EQ-5D), and fear of falling (FES). They also underwent a physical pre-trial gait (TUG) and balance (BERG) assessment. Note that, a MMSE score greater than 25 represents no cognitive impairment; a EQ-5D VAS score of 0 and 100 represents ‘the worst and the best imaginable health’, respectively; a FES score lower or equal to 70 indicates fear of falling; a TUG score greater than 12 indicates the individual is at risk of falling; and a BERG score below 46 indicates a risk of falling.

### Experimental procedures

Each participant underwent a walking trial conducted indoors on a treadmill. For preferred walking speed (PWS) determination^13^, participants were asked to start walking on the treadmill and indicate when comfortable with the treadmill’s speed, while the treadmill speed was gradually increased in increments of 0.1 km/h. Once comfortable, additional increments of 0.1 km/h were added until they indicated it was becoming “too fast to be comfortable”. The same procedure was conducted in the opposite direction, until the participant referred it to be “too slow to be comfortable”. The average of the two measures was considered as PWS. The participants then continued walking for 3 min at the PWS speed for familiarization. After a minimum 5-min rest, participants were then asked to walk at their previously determined PWS for 12 consecutive minutes looking straight ahead, while ignoring eventual visual or auditory distractions. A miniaturized triaxial accelerometer (Plux Biosignals, Portugal), placed at the lateral malleoli, was used to determine gait events. Acceleration data was collected at 1000 Hz. Electromyography (EMG) from the Gastrocnemius Medialis (GM) was collected resorting to a telemetric system (Plux, Lisbon, Portugal) at 1000 Hz. First, impedance was minimized by shaving and cleaning the skin with an alcohol solution. Following SENIAM project recommendations, disposable pre-gelled Ag/AgCl electrodes with inter-electrode distance of 20 mm were placed^14^. To avoid movement interference, the wire was securely fixed with tape. Acceleration and EMG signals were synchronized.

For analysis purposes, the first 15-s were removed to avoid any transient effects with the adjustment to the treadmill’s speed. A 4th order, zero lag low-pass Butterworth filter with a cutoff frequency of 20 Hz was applied to the accelerometer signal. Filtering cutoff frequency was defined according to Winter^15^. A custom Matlab® code was used to determine inter-stride intervals (ISIs), identified as the time difference between two consecutive heel strikes of the same foot. Raw EMG signals were band-pass filtered (20–500 Hz), full-wave rectified and smoothed with a low-pass filter (12 Hz, 4th order Butterworth), following the 2017 recommendations from the International Society of Electromyography and Kinesiology. Afterwards, the peak maximum from each gait cycle was found and the time difference between two consecutive peaks was determined: inter muscle peak intervals (IMPIs). Visual inspection of the peaks' identification was conducted both on the acceleration signal and on the inter stride intervals time series. The coefficient of variation (CV) and the fractal scaling exponent α were calculated for each ISIs and IMPIs time series. The CV was used as a measure of the magnitude of variability, while the fractal scaling exponent α was used as a measure of the temporal structure of variability. Detrended Fluctuation Analysis (DFA) was used to determine the fractal-scaling exponent α for ISIs and IMPIs time series^16–20^. Window sizes of 16 to N/9 were used, where N is the length of the data^21^. All methods were carried out in accordance with relevant guidelines and regulations.

### Statistical analysis

Analysis was performed using *jamovi* (version 1.6) with the level of significance set a priori to 0.05. Descriptive means, standard deviations and confidence intervals were calculated for ISIs and IMPIs. The assumption of normality was tested through Shapiro Wilk’s Test. The Pearson’s Correlation coefficient was used to study the association between α-ISIs and α-IMPIs. The correlation coefficient was interpreted according to Cohen^22^: small [0.1:0.3[, moderate [0.3:0.5] or strong [0.5:1.0]. To assess the differences between ISIs and IMPIs, Sample’s T-tests were used. In the case normality was not verified, Wilcoxon Signed Rank Test was used.

### Ethics statement

The studies involving human participants were reviewed and approved by the Ethics Committee of the Faculty of Human Kinetics, University of Lisbon. The participants provided their written informed consent to participate in this study.

## Results

### Health-related questionnaires

Overall, the participants revealed high cognition level (MMSE), general high levels of perceived quality of life and health status (EQ-5D-VAS), high levels of balance confidence (FES and BERG) and, also, general good level of physical performance, as presented in Table 1.

**Table 1:** Demographics and sample’s features (N = 21). Data are presented as Mean ± Standard Deviation.

	M ± SD
Age (yrs)	70.3 ± 4.5
Height (m)	1.68 ± 0.07
BMI	26.7 ± 4.0
MMSE	29.0 ± 1.0
FES	98.2 ± 4.7
EQ5D-VAS	87.8 ± 7.2
TUG (s)	7.2 ± 1.3
BERG	55.1 ± 1.3

*MMSE* mini mental state examination, *FES* Falls Efficacy Scale, *EQ-VAS* overall self-rated health status, *TUG* timed up and go, *BERG* Berg Balance Scale.

### Gait-related parameters

**Figure 1:**
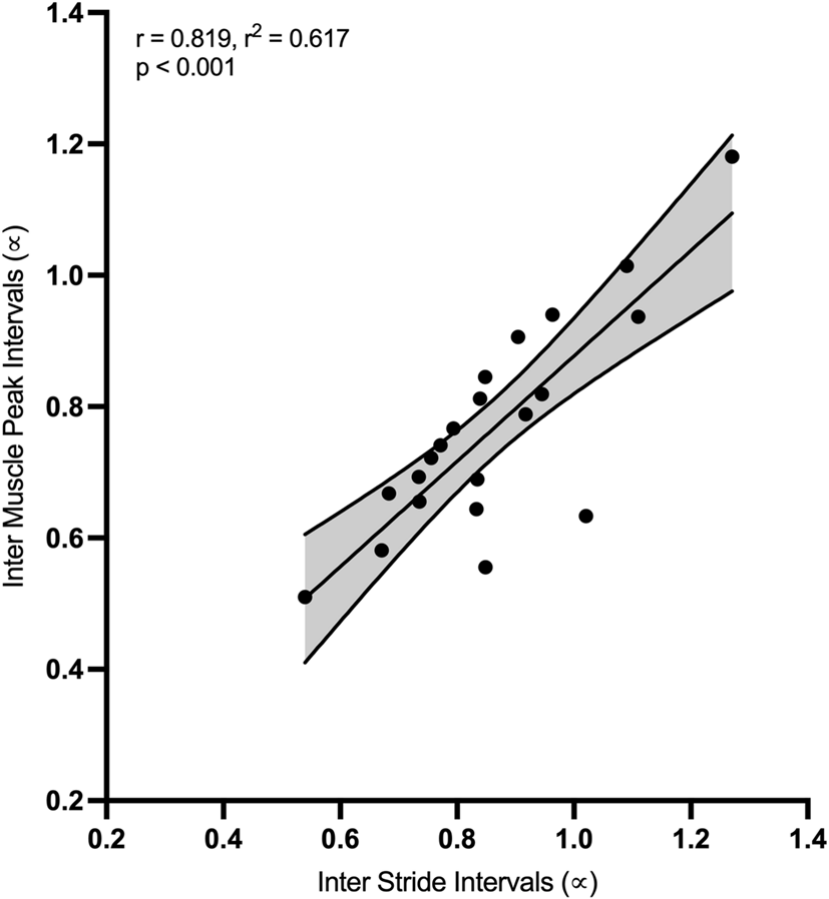
The correlation between Inter Muscle Activity Intervals (α-IMPIs) and Inter Stride Intervals (α-ISIs). The individual data points represent each participant value.

A significant, strong positive, correlation was observed between α-ISIs and α-IMPIs (*r* = 0.819, *r*^2^ = 0.671, *p* < 0.001), as depicted in Fig. 1.

**Figure 2:**
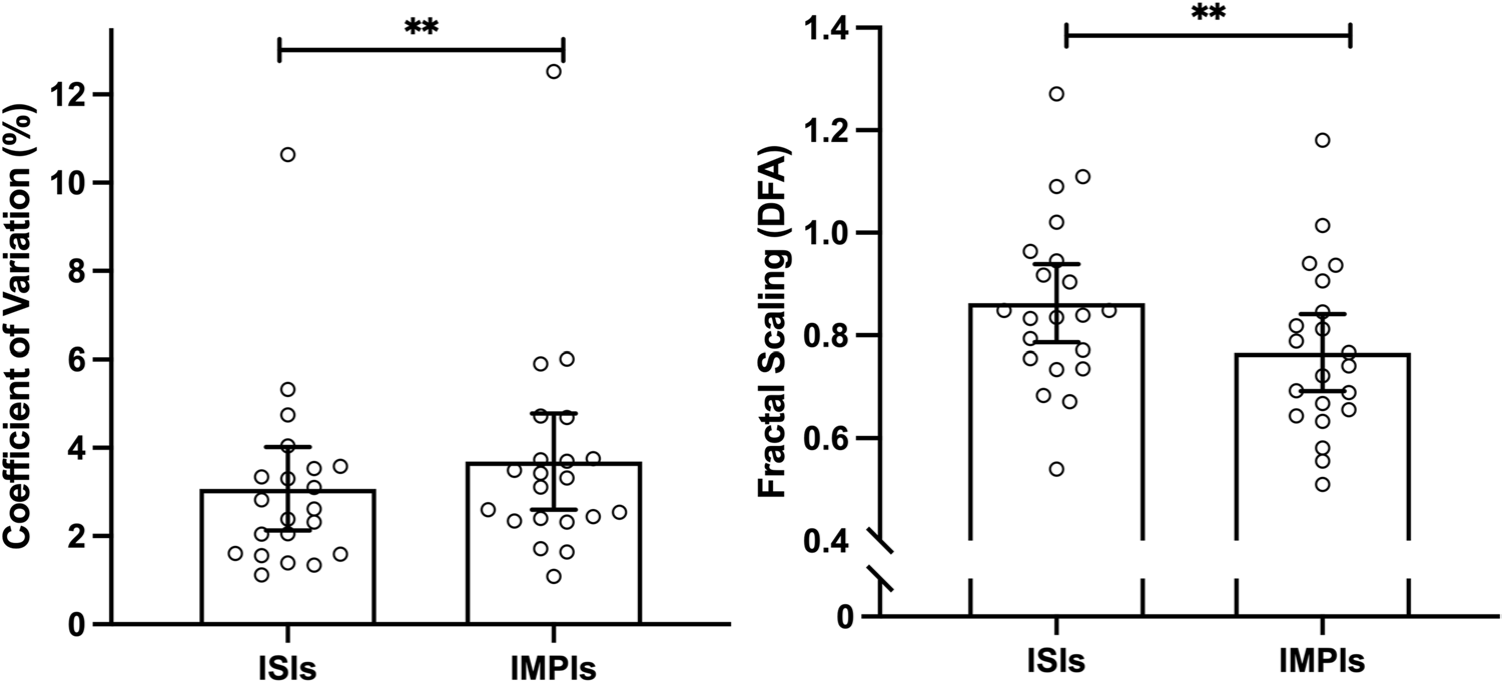
The mean values of Coefficient of Variation (%) and Fractal Scaling of Inter Stride Intervals (ISIs) and Inter Muscle Activity Intervals (IMPIs). Data are presented as Mean ± 95% of Confidence Intervals. The individual data points represent each participant value. ** indicates *p* < 0.01.

Regarding the comparison between α-ISIs and α-IMPIs, we have found a significant difference (0.86 ± 0.16 and 0.77 ± 0.16, respectively, t = 4.393, *p* < 0.001, *d* = 0.959), as represented in Fig. 2. In addition, we also observed significant differences between CV-ISIs and CV-IMPIs (3.07 ± 2.03% and 3.69 ± 2.34%, respectively, Z = − 3.180, *p* < 0.001, *r* = − 0.49). Conversely, no differences were observed between mean-ISIs and mean-IMPIs (t = − 0.103, *p* = 0.919, *d* = − 0.023; 1.22 ± 0.15 s and 1.22 ± 0.15 s, respectively).

## Discussion

This study aimed to investigate if the temporal structure of gait variability patterns of older adults correlates with the temporal structure of the *gastrocnemius medialis* (GM) muscle activity variability patterns. In addition, we also investigated if the temporal structure of the two systems differed between each other. We hypothesized a positive significant correlation, and that no differences would exist between the two systems. Our hypotheses were partly supported. We did find a strong positive correlation between the temporal structure of gait patterns and muscle activity, suggesting a close interaction between the mechanical output and the neuromuscular processes. However, contrary to what we anticipated, we found that mechanical and muscle activity patterns presented differences in their temporal structure.

This study shows a similar behaviour between the patterns of two systems (gait and muscle activity). In other words, those individuals that presented more randomness in their gait patterns (i.e., lower fractal scaling values), also presented greater randomness in their muscle activity patterns. Although out of the scope of the present study, our findings suggest that fractal scaling can be seen as a surrogate biomarker of health. In addition, the present study findings also support the multi-level interaction in the occurrence of adaptable flexible behaviors. Thus, our study corroborates Goldberger and colleagues’ theoretical framework of many levels of interactions within a functioning healthy neuromuscular system^5^. Indeed, functional gait relies on complex processes involving the neuromuscular system^23–25^. This system depends on the coordination of processes involving sensory integration and executive function. Muscles are activated by the motor neurons which, in turn, receive rhythmic activity generated from specialized neurons in the spinal cord and brainstem. The cerebral cortex integrates input from proprioceptive, visual, and vestibular systems; additional input is received from the cerebellum, brainstem, basal ganglia, and afferent neurons carrying proprioceptive signals from muscle stretch receptors^24^. The interaction between these structures and systems allows individuals to walk safely and be able to adapt to environmental constraints.

Previous research has shown that gait variability can be manipulated through different strategies such as interpersonal coordination by walking arm-in-arm^7^, tai-chi practice^26^ and synchronizing to a fractal-like metronome^8,9,27^. While Almurad and colleagues’ experiment was based on a training program involving older adults walking synchronized with young companions resulting in a variability matching effect^7^, Vaz and colleagues’ approach consisted of gait trials where old adults walked at their self-selected speed while synchronizing to a fractal-like visual stimulus^8^. Hove and colleagues demonstrated that an “interactive” auditory stimulus based on non-linear oscillators restored the locomotor fractal properties in Parkinson’s disease patients^9^, and Marmelat and colleagues also had Parkinson’s disease patients synchronizing to fractal auditory stimuli^27^. Regardless the approach, it appears neuromuscular activity is being somehow affected, as there are observable modifications in the gait patterns. Therefore, it is reasonable to suggest that changes promoted by, for example, cues synchronization, can be expanded to the neuromuscular processes and not limited to mechanical changes at the gait pattern level. The mechanism(s) responsible for the fractal properties of stride-to-stride intervals in healthy individuals remain unknown and may be a consequence of peripheral input or related to higher nervous system centers that control the walking rhythm^28^. Although speculative, considering these strategies can induce the restoration of muscle activity patterns, this could mean that it is the flexibility of the nervous system to adjust, that will determine gait patterns adaptability. In other words, changes in the temporal structure of muscle activity patterns can potentially precede the mechanical gait patterns’ temporal structure.

Despite finding a strong positive correlation between gait and muscle activity temporal structures, our results showed significant differences between the two (α-ISIs = 0.86; α-IMPIs = 0.77). Considering previous research, our mean α-ISIs (0.86) can be seen as relatively high for older adults. This can be explained by the health status of our sample, who present a good level of quality of life and confidence (Table 1). This is further observed on the individual data distribution depicted in Fig. 2. Similar to the differences observed between α-ISIs and α-IMPIs, we also observed differences at the magnitude of variability level (CV-IMPIs = 3.69%; CV-ISIs = 3.07%). These latter findings can be attributed to several causes. At the peripheral level, the ankle/foot complex joint is a functional unit essential in the neuromotor control of gait. Plantar flexors are responsible for approximately 70% of the ankle’s muscle activity^29^, supporting the body weight and insuring the advance of the centre of mass during the propulsion phase in the gait cycle^30,31^. Thus, the gastrocnemius muscle has a critical role in the propulsion phase, which explains the selection of such muscle to the current study. However, we are certainly aware that despite playing a central role in the propulsion action, the gastrocnemius muscle is not the only relevant muscle during the gait cycle. If the remaining muscles that were not considered in this study were accounted for, the mean value of α-IMPIs would likely have been closer to the mean value of α-ISIs. Furthermore, the nature of the collected signals must be taken into consideration when interpreting the results. The EMG signal presents higher level of experimental noise, despite being filtered. It is, therefore, possible that the nature of the signal and the pre-processing procedures can partly explain the differences observed between ISIs and IMPIs. To address these limitations, future studies should add more muscles into the analysis to interpret these observed differences further and robustly between ISIs and IMPIs.

The present study brought to evidence the multilevel interaction between elements/systems in human physiology. We have showed that the patterns of the temporal structure of the fluctuations in the gait are related with those at the muscle activity level. To further support the theoretical framework that suggests this multilevel dynamic interaction, future research should include additional biological rhythms. Interestingly, it has been suggested that neurons in the brain also interact and communicate non-linearly and through paths and connections over multiple timescales^32^ ranging from sub milliseconds (transmission of neural impulses) to hours (circadian rhythms). Consequently, the dynamics of spontaneous healthy brain activity are complex and contain information with fractal-like patterns that are self-similar over multiple scales of time^33^. In addition, Kamal and colleagues strengthened this concept by showing that healthy variable patterns are present in the brain and heart when participants walk on a fractal-like path^10,11^. Thus, consider additional biological rhythms will strengthen the multilevel dynamic interaction hypothesis. Furthermore, future research should consider how such relationships could be affected under manipulations of variables that could affect their patterning. Will they change in a similar fashion or not?

The understanding of the physiological processes underlying the interdependency and interaction of the neuromuscular system’s components might revolutionize the strategies used in gait rehabilitation. Although further investigation to support our findings is necessary, the strong positive correlation identified between gait and muscle activity patterns in older adults could support innovative clinical gait rehabilitation approaches, such as the incorporation of variability with gait retraining modalities.

## Conclusion

This study shows a strong positive correlation between the temporal structure of gait and muscle activity variability patterns of the older adults, indicating a close interaction between these two elements of the human neuromuscular system.

## Data Availability

The datasets generated for this study are available on request to the corresponding author.
